# Artificial Intelligence in Public Health: Bridging Today’s Trends with Tomorrow’s Possibilities

**DOI:** 10.3390/bioengineering12060559

**Published:** 2025-05-23

**Authors:** Daniele Giansanti, Giovanni Costantini

**Affiliations:** 1 Centre Tisp, Istituto Superiore Di Sanita, 000161 Rome, Italy; 2 Department of Electronic Engineering, University of Rome Tor Vergata, 00133 Rome, Italy; giovanni.costantini@uniroma2.it

Driven by the unprecedented challenges of the COVID-19 pandemic, the healthcare sector has witnessed remarkable—and at times sometimes overwhelming—advancements in the role of artificial intelligence (AI) [1]. The pandemic underscored the urgent need to enhance health systems and better manage healthcare data, prompting a surge in the development and application of AI technologies [2]. From tracking disease outbreaks to optimizing patient care and managing hospital resources, AI has emerged as a critical tool in reshaping health systems, especially in times of crisis [3].

The pandemic has highlighted both the potential and the complexities of integrating AI into healthcare [4]. The rapid shift toward digital health solutions has accelerated the adoption of technologies that were previously seen as futuristic, making them vital to the global response to the health crisis [5]. Innovations such as wearable health monitoring, big data analysis, and robotic surgery have played key roles in addressing the immediate demands of the pandemic [6,7]. The range of AI applications [8] that emerged during this period is broad, including diagnostics for organ and tissue conditions, care robotics for rehabilitation and disability support, biomedicine encompassing genetics and modeling, and precision medicine that tailors treatments to individuals’ genetic profiles.

As Henry Ford famously stated, “Real progress happens only when the advantages of a new technology become available to everybody”. The COVID-19 pandemic has brought this statement into sharp focus, as the world has recognized the importance of ensuring that the benefits of AI and digital health advancements are accessible to all, regardless of geography or socioeconomic status [9]. AI in healthcare is not merely a technological leap; it represents a critical pathway toward equitable healthcare for all.

The integration of AI in healthcare systems is designed to enhance care quality and promote equity. Its influence will extend far beyond immediate health crises, shaping the future of disease prevention, personal care delivery, and the management of healthcare systems. In particular, AI holds the potential to impact the prevention of diseases at both the individual and societal levels, offering insights into disease prediction, monitoring, and personalized treatment options. The integration of AI with other technologies and solutions [8,9], such as robotics and virtual and augmented reality, could create a new era of accessible virtual healthcare services and further enhance the precision and safety of robotic surgeries.

To address these challenges, a comprehensive Topic [10] was proposed, which included research published in several prominent journals, such as *Healthcare*, *Applied Sciences*, *AI*, Bioengineering, *JCM*, and *IJERPH*. This Topic aims to provide a thorough exploration of AI’s role in transforming healthcare systems, spanning from scientific advancements to real-world applications. It also focuses on critical ethical and training considerations, encouraging researchers and practitioners to consider how AI can be deployed responsibly and effectively. In this editorial, we will present the results and key findings from the research collected in this Topic, highlighting the significant progress made in AI applications and their implications for the future of public health.

Thanks to the contribution of numerous international research groups, this Topic has reached 26 publications in addition to this concluding editorial. As shown in Figure 1, sixteen articles (AR) [11,12,13,14,15,16,17,18,19,20,21,22,23,24,25,26]; seven reviews including two systematic reviews [27,28,29,30,31,32,33]; one technical note (TN) [34]; one perspective (P) [35]; and one editorial (ED) [36] presenting the project have been published.

*Contributions of Studies*

Table 1 synthesizes the contributions of the studies included in this Topic.

The integration of artificial intelligence (AI) and machine learning (ML) has become increasingly important in improving healthcare systems. Studies [11,12,13,14,15,16,17,18,19,20,21,22,23,24,25,26] illustrate diverse applications across various domains, from predicting patient outcomes to optimizing diagnostic accuracy and enhancing healthcare management.

One significant area of development is the use of AI for predicting patient outcomes. For example, ref. [12] investigates delayed treatment behavior in oral cancer patients in Western China, using ML models to predict delays and identify risk factors. Similarly, ref. [13] applies ML models to predict mortality risk in emergency department patients based on routine clinical data, aiming to enhance patient triage and prioritization. Both studies demonstrate how AI can support more effective and timely predictions, potentially saving lives by allowing clinicians to intervene earlier. These studies highlight the growing role of AI in supporting medical decision-making and resource allocation.

Medical image analysis also shows promising results, particularly for detecting and classifying diseases. For instance, ref. [15] explores the use of radiomics and AI to predict progression-free survival in high-grade glioma patients, while ref. [22] introduces a dual deep convolutional neural network (DCNN) model to classify brain tumors in MRI scans. By analyzing medical images using AI, clinicians can obtain more precise, personalized treatment plans. Likewise, ref. [24] enhances brain tumor detection through an improved Fuzzy C-Means algorithm, further underscoring AI’s potential in improving diagnostic accuracy and early detection. AI-powered diagnostic tools are crucial in improving patient outcomes by enabling earlier intervention and more accurate treatment strategies.

The role of AI in mental health and well-being is another key area that is explored. The authors of [14] propose an AI system to automate mental health evaluations and generate personalized medical advice, highlighting AI’s potential in streamlining assessments and offering tailored treatment options. Moreover, ref. [18] investigates the use of a virtual companion for people with dementia in long-term care, which uses AI-driven interactions to reduce loneliness and promote meaningful social engagement. Both studies emphasize AI’s ability to provide personalized, patient-centered care in mental health.

AI’s impact on public health surveillance is also noteworthy. The authors of [11] explore the use of AI and deep learning models like YOLOv8 for real-time tracking of disease transmission in indoor spaces, improving public health monitoring. This is complemented by [19], which examines the impact of COVID-19 on emergency medical services in Kazakhstan, revealing shifts in healthcare service demand during and after the pandemic. Both studies demonstrate how AI can optimize healthcare responses during health crises and assist in epidemiological monitoring.

Additionally, AI for resource management in healthcare systems is addressed in studies like [16], which uses ML models to predict the length of stay in pediatric intensive care units, and [17], which applies ensemble ML models to predict injuries from the National Electronic Injury Surveillance System dataset. These studies highlight how AI can improve operational efficiency, resource allocation, and decision-making in healthcare settings.

AI’s role in improving diagnostic accuracy is examined in [20], which compares the diagnostic accuracy of Google and ChatGPT 3.5 for urological conditions, showing that ChatGPT 3.5 outperforms Google for common conditions. Similarly, ref. [25] discusses AI’s role in summarizing scientific research for public health applications, demonstrating its potential in assisting researchers with literature reviews and scientific writing. The authors of [26] present the DLShelper tool, which enhances the segmentation of lesions in CT images, aiding in more accurate COVID-19 diagnoses.

Overall, these studies collectively demonstrate AI’s transformative potential across various healthcare domains, from improving diagnostic accuracy and predicting patient outcomes to optimizing resource management. As AI continues to evolve, it offers promising solutions to address challenges in patient care, health management, and public health, providing a foundation for more efficient and personalized healthcare systems.

*Contribution of Review Studies*

Table 2 presents a summary of the review studies included in this Topic. 

These studies explore a range of AI applications in different healthcare fields, demonstrating the transformative potential of these technologies across diverse medical specialties.

The review on artificial intelligence in cytopathology [27] provides an umbrella review that assesses the integration of AI into cytopathology, focusing on how it can improve diagnostic accuracy and operational efficiency. AI’s ability to automate processes, reduce diagnostic errors, and enhance patient outcomes is explored, although challenges such as high implementation costs and algorithmic biases remain.

The systematic review on Generative Adversarial Networks (GANs) in head and neck surgery [28] investigates how GANs, which can generate new data from existing data, are used in craniofacial surgery. It identifies their potential to enhance diagnostic imaging, surgical planning, and post-operative predictions, particularly in treating complex conditions like craniosynostosis and bone defects.

Mobile and domiciliary radiology with AI integration is investigated in the overview reported in [29] that discusses the growing role of mobile radiology and AI in improving healthcare access, especially in rural and underserved areas. AI-powered diagnostic tools and mobile X-ray units, particularly in breast cancer screening, have been transformative in domiciliary radiology, making it an essential service post-COVID-19.

The systematic review on AI chatbots in women’s health [30] instigates how AI-powered chatbots are used to improve mental health, health behaviors, and preconception care among women. This study emphasizes the effectiveness of these chatbots in addressing mental health issues such as anxiety and depression, and how they offer a scalable, cost-effective solution for women’s health, particularly in remote or underserved areas.

The review on AI and language models in cardiology [31] explores the use of generative AI, such as ChatGPT-4, in cardiology, focusing on how these tools can assist in diagnosing heart disease, recommending treatment plans, and automating administrative tasks. It underscores the benefits of AI in improving efficiency and diagnostic accuracy, but also highlights concerns like outdated data and the loss of human empathy in patient care.

The review on AI in teledermatology [32] examines how AI is enhancing teledermatology, particularly in remote skin condition diagnosis. This review highlights AI’s role in increasing accessibility, reducing healthcare costs, and improving diagnosis efficiency. However, it also calls for better app design, cybersecurity, and regulatory frameworks to ensure that AI applications are safely implemented.

The review on machine learning in domestic violence detection [33] explores how machine learning algorithms are being used to detect early signs of domestic violence by analyzing digital data. This review identifies the potential of ML in predicting domestic violence and enhancing early intervention, but it also acknowledges challenges related to data quality and algorithmic biases.

Overall, these studies collectively emphasize how AI and machine learning are revolutionizing various aspects of healthcare, from diagnostics and treatment planning to improving access to care. The common message across all these studies is that AI is becoming a powerful tool in medical fields, enhancing efficiency, improving patient outcomes, and addressing healthcare inequalities. However, challenges such as data privacy, algorithmic bias, and integration with existing systems need to be carefully addressed to fully realize the potential of these technologies.

*Contribution of Other Studies*

Table 3 presents a summary of other studies published in this Topic.

The two studies presented in Table 3 focus on the application of AI in healthcare and environmental research.

Machine learning for effective dose calculation is investigated in a technical note [34], exploring how ML algorithms can predict organ doses and effective dose conversion coefficients (DCCs) for anthropomorphic phantoms. Traditionally, these calculations are performed using Monte Carlo methods, which are time-consuming. By training ML models like XGB, GB, and Extra Trees regressor on Monte Carlo datasets, the authors demonstrate that ML can predict doses efficiently, providing a faster alternative. This approach offers a solution to the time and expertise required for traditional methods, contributing to personalized dosimetry in radiation protection.

Neural network models for causal variable analysis in medical and climatic studies are investigated in a perspective [35], presenting a neural network-based method to analyze non-linear systems with small datasets, focusing on identifying causal variables in medical and climate research. The methodology helps predict future trends, particularly in cancer studies. This approach is valuable for medical research with limited data and provides insights into causal relationships. This study’s contribution lies in improving predictions and understanding complex factors, which could influence cancer treatment and other medical applications. Both studies demonstrate the potential of AI to improve efficiency and accuracy in healthcare and research, offering practical solutions and insights for better decision-making and predictions.

*Conclusions and message for future work*

The studies reviewed here highlight the increasing role of artificial intelligence (AI), and, in particular, machine learning (ML), in advancing healthcare practices, particularly in diagnostic accuracy, treatment planning, and personalized medicine. In various medical fields, such as radiology, cardiology, dermatology, and domestic violence detection, AI and ML are improving the efficiency, accuracy, and accessibility of healthcare services. These innovations not only enhance diagnostic precision but also contribute to reducing operational costs, optimizing workflows, and fostering personalized approaches to care, especially for underserved populations.

A recurring theme across these studies is the importance of integrating AI and ML technologies into existing healthcare frameworks. This involves addressing challenges related to data quality, algorithmic biases, and clinical workflow integration, ensuring that these technologies are both clinically and ethically appropriate. Additionally, the need for rigorous validation, regulatory frameworks, and ongoing assessment remains central to their responsible use in healthcare.

The studies on mobile and domiciliary radiology and AI chatbots for women’s health, in particular, highlight the potential of these technologies to bridge gaps in healthcare access. By leveraging mobile devices and telemedicine platforms, AI innovations can improve healthcare access in rural or underserved regions, thereby improving health outcomes and promoting equity.

Despite promising results, significant challenges remain. The adoption of AI and ML tools in healthcare requires careful consideration of technical constraints, such as the need for larger and more diverse datasets, and ethical concerns, including data privacy, security, and bias mitigation. Comprehensive training for healthcare professionals is also essential to ensure they can effectively use AI tools in clinical decision-making.

Future work should focus on the following areas:

- Expanding datasets to improve the accuracy and generalizability of AI/ML models across different healthcare populations and settings.
- Developing interdisciplinary frameworks for better integration of AI solutions into clinical contexts, addressing ethical concerns, and ensuring equity.
- Exploring AI’s potential in less-explored areas, such as mental health, long-term disease management, and healthcare logistics.
- Enhancing regulatory frameworks to keep pace with the rapid development of AI/ML technologies, ensuring safe and responsible implementation.
- Promoting the development of user-friendly AI tools that clinicians can adopt without disrupting existing workflows.

In conclusion, while AI and ML have the potential to revolutionize healthcare, their integration must be approached with care, balancing technological innovation with patient safety, ethics, and equity. Future work should focus on overcoming existing barriers and optimizing AI solutions for real-world clinical applications.

## Figures and Tables

**Figure 1:**
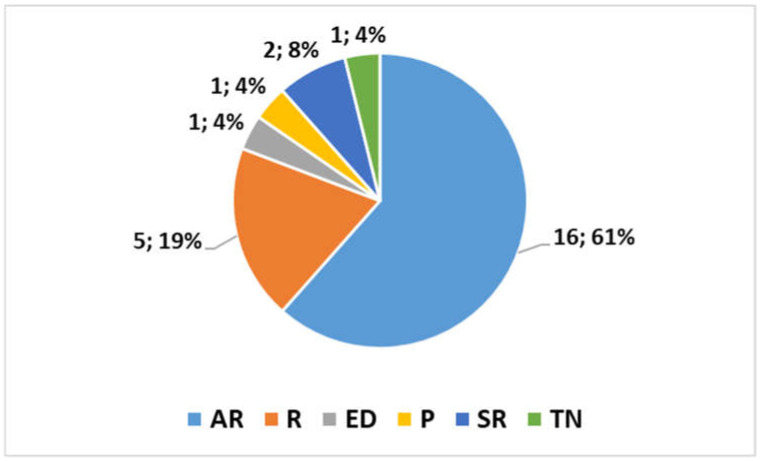
Number and types of publications contributed to the Topic (articles—AR, reviews—R, technical notes—TN, perspectives—P, systematic reviews—SR, editorials—ED).

**Table 1:** Summary of the articles published in this Topic.

Study	Focus	Brief Summary	Contribution
[11]	Indoor transmission of respiratory diseases and real-time contact rate analysis	This study addresses the increasing concern about airborne diseases spreading in indoor spaces, particularly in settings like offices, schools, and hospitals. It introduces an advanced software tool powered by YOLOv8, a state-of-the-art deep learning model, to accurately detect and track individuals from video streams. The tool uses dynamic circular buffer zones and a 2D projective transformation to overlay digital coordinates of the physical environment, visualizing heatmaps of spatial contact patterns and quantifying contact events. This method provides a more precise and automated alternative to traditional manual contact tracing and counting techniques.	This study introduces a transformative shift in public health surveillance by integrating real-time tracking and AI to model disease transmission more accurately and efficiently. The system’s ability to offer precise, dynamic tracking within enclosed spaces will significantly enhance epidemiological studies, improve the management of disease outbreaks, and provide public health officials with a robust tool to develop non-pharmaceutical interventions based on real-time, spatially detailed data.
[12]	Delayed medical treatment behavior in oral cancer patients in Western China and machine learning for prediction	This research investigates the social, economic, and health factors contributing to delays in seeking medical treatment among oral cancer patients in Western China. A combination of logistic regression and machine learning models (such as nearest neighbors, SVM, and random forest) is applied to identify key risk factors and predict the likelihood of delayed treatment. This study compares the behavior of patients before and after the lifting of COVID-19 restrictions, offering insights into the pandemic’s impact on medical delays.	This study provides a unique and large-scale analysis of delayed treatment behavior, with a focus on how family dynamics, social support, and external factors like the pandemic influence patients’ decision-making. By developing a predictive model, it offers valuable insights into healthcare policies and early intervention strategies, especially in regions with limited healthcare access. It underscores the importance of social support, highlighting the role of family structures in reducing treatment delays and potentially improving outcomes.
[13]	Short- and long-term mortality prediction in emergency department patients using clinical data	This study focuses on creating predictive models for mortality risks in patients admitted to emergency departments (EDs) by using minimal, routine clinical data—specifically, blood biochemistry results from a single admission sample. This study uses datasets from several large Danish hospitals, applying machine learning models to predict patient mortality within 10, 30, 90, and 365 days post-admission. The goal is to enhance patient triage and prioritization, particularly in settings where timely decisions can save lives.	The contribution lies in proving that routine biochemistry tests, often performed at admission, can be leveraged to accurately predict both short-term and long-term mortality. This study proposes a simplified yet highly effective tool for mortality prediction, which could become an integral part of emergency care workflows, helping clinicians make better decisions regarding patient management and resource allocation in EDs.
[14]	AI-assisted mental health evaluation and personalized medical advice generation	This paper proposes an integrated AI system that combines finite state machines, semantic matching algorithms, and medical knowledge graphs to improve mental health assessments. By automating the collection of patient data and conducting preliminary evaluations, the tool optimizes decision-making. It then generates personalized medical advice based on deep learning techniques such as RoBERTa for semantic matching, ensuring accuracy in recommending treatment options.	This study showcases how AI can significantly improve mental healthcare by streamlining the assessment process and providing personalized advice. The integration of AI-driven diagnostics with medical knowledge graphs leads to more accurate recommendations, ensuring efficient use of medical resources. The findings have the potential to reduce the diagnostic burden on mental health professionals and provide a personalized care experience for patients.
[15]	Radiomics and AI in predicting progression-free survival in high-grade glioma patients	This study explores the use of radiomics (the extraction of quantitative features from medical images) combined with AI algorithms to predict progression-free survival (PFS) in patients with high-grade gliomas, a type of brain cancer. The research focuses on preoperative MRI scans, analyzing 109 radiomic features along with clinical factors like age, sex, and tumor location to develop predictive models.	This research contributes to personalized oncology by demonstrating how AI models, when integrated with radiomic features, can provide accurate predictions of progression-free survival in glioma patients. These insights can assist in stratifying patients based on their risk of tumor recurrence, potentially guiding tailored treatment plans. The study emphasizes the importance of early prediction in managing high-grade gliomas and improving patient outcomes through more individualized treatment strategies.
[16]	Machine learning for predicting length of stay in pediatric intensive care units (PICU)	This study investigates the application of machine learning models to predict the length of stay (LOS) in pediatric intensive care units (PICUs). This research uses data from over 100 North American PICUs, applying algorithms such as support vector machine, Gradient Boosting, and Recurrent Neural Networks (RNNs) to predict patient outcomes at various thresholds (24, 36, 48 h, etc.). The primary goal is to improve resource management and optimize staffing within PICUs.	This study highlights how machine learning can outperform traditional methods of predicting PICU LOS, with models like Gradient Boosting and RNNs achieving higher accuracy and balanced performance. These models provide critical insights that can be used to better allocate resources, optimize staffing, and improve patient care. The findings suggest that further refinement of machine learning models could enhance their applicability in clinical settings, improving operational efficiency in pediatric intensive care units.
[17]	Ensemble machine learning models for injury prediction	This study analyzes the use of ensemble machine learning (ML) models to predict injury codes from the National Electronic Injury Surveillance System (NEISS) dataset. Four ensemble models were tested: random forest (RF) + logistic regression (LR), K-Nearest Neighbor (KNN) + RF, LR + KNN, and a combined LR + RF + KNN approach. The KNN + LR ensemble model achieved 90.47% accuracy for the top injury code, while the combined KNN + LR + RF model excelled with 99.50% accuracy for predicting the top two injury codes.	This study demonstrates how ensemble ML models, particularly the probabilistic framework, can enhance the accuracy of unstructured narrative classification, especially when dealing with imbalanced data. The findings underscore the potential of these models to improve decision-making in public health, especially in injury classification systems.
[18]	Virtual companion for people with dementia in long-term care	This study focuses on developing a virtual companion for people living with dementia (PLWD) in long-term care (LTC). The system, delivered via a head-mounted virtual reality display, initially prompted conversation and reminiscence, and later used advanced AI to allow more nuanced interactions. Testing showed that while the system faced challenges in early stages, the improved AI in later stages led to more meaningful interactions. Despite some issues like speech recognition and display weight, the system was found to be enjoyable for many users.	This research illustrates the application of virtual reality and AI to provide an engaging, socially interactive tool for PLWD in LTC, promoting meaningful conversations and reducing loneliness. It lays the groundwork for future enhancements, emphasizing the need for innovations in both hardware and software to improve user experience.
[19]	Impact of COVID-19 on emergency medical services in Kazakhstan	This retrospective study examines the structure and frequency of ambulance calls in Kazakhstan from 2019 to 2023, focusing on changes during and after the COVID-19 pandemic. The analysis found significant fluctuations in urgency category IV calls, with decreases during the pandemic due to quarantine restrictions and a subsequent increase in the post-pandemic period.	The research provides valuable insights into the dynamics of EMS call volumes in response to the COVID-19 pandemic. It highlights the shifts in healthcare service demand and the long-term impact on emergency medical systems, particularly in less urgent cases. This study offers critical data for improving future emergency healthcare responses during pandemics.
[20]	Comparison of diagnostic accuracy between Google and ChatGPT 3.5 for urological conditions	This study compares the diagnostic accuracy of two freely available platforms, Google and ChatGPT 3.5, for identifying urological pathologies. A sample of 60 clinical cases was used to assess each platform’s performance. ChatGPT 3.5 showed superior accuracy, especially for common urological conditions, while Google was less reliable, particularly for rare disorders.	The findings highlight the increasing role of AI platforms in medical diagnosis, showing that ChatGPT 3.5 outperformed Google in providing accurate diagnoses, particularly in common urological conditions. This research sets the stage for exploring the use of AI tools in clinical practice, though further improvements are necessary for rare condition diagnosis.
[21]	Radiomics and machine learning for predicting survival in NSCLC patients	This retrospective study investigates the use of radiomics and machine learning to predict survival in non-small cell lung cancer (NSCLC) patients using FDG PET/CT imaging. It evaluates a radiomic model, incorporating tumor stage, SUVmax, and selected radiomic features like NGTDM coarseness, and compares it with a traditional survival model based only on tumor stage and SUVmax.	This study demonstrates that integrating radiomic features with traditional clinical data can offer similar predictive power for survival in NSCLC patients. While the radiomic model’s accuracy was comparable to traditional methods, the findings suggest a potential for more precise, personalized prognostic models in the future.
[22]	Deep learning for brain tumor detection in MRI scans	This research introduces a novel dual deep convolutional neural network (DCNN) model designed for classifying brain tumor MRI scans. By using two deep learning models (InceptionV3 and DenseNet121) for feature extraction, this study achieves high performance in classifying brain tumors as cancerous or non-cancerous. It compares this dual DCNN approach against other popular deep learning architectures, showing superior performance.	This study presents a highly effective AI model for brain tumor detection, outperforming traditional deep learning methods in terms of accuracy, precision, and recall. This approach highlights the potential for deep learning models to revolutionize early cancer detection through automated analysis of medical images, which could greatly enhance diagnostic efficiency and outcomes.
[23]	Artificial intelligence for early detection of hemorrhage and organ dysfunction during delivery	This study leverages machine learning to establish numerical criteria for identifying organ dysfunction in cases of massive hemorrhage during delivery. The research uses a dataset of 107 deliveries with >2000 mL blood loss from nine national perinatal centers in Japan. By analyzing coagulation factors like fibrinogen and FDP, this study identifies thresholds for predicting hematuria development, which can indicate organ dysfunction. It applies six different machine learning methods, with the support vector machine being the most effective in determining the relevant clinical criteria.	This study presents a novel AI-driven method to detect organ dysfunction in massive hemorrhage cases during delivery. By providing more precise numerical thresholds and combining machine learning with clinical coagulation data, the method could significantly improve the early identification of severe complications in obstetric care, leading to quicker interventions and better patient outcomes.
[24]	Advanced Fuzzy C-Means segmentation for MRI brain tumor detection	This research focuses on improving the Fuzzy C-Means (FCM) segmentation algorithm for MRI images to enhance brain tumor classification. This study integrates feature selection based on tumor shape, texture, and color, which reduces complexity and increases classification accuracy. It then uses the Extreme Learning Machine (ELM) classifier to segment and categorize brain tumors. The performance of this enhanced model is compared with existing methods, and it consistently demonstrates superior accuracy, particularly in detecting gliomas.	The improved FCM-based model delivers better performance for tumor classification in MRI scans, with notable improvements in accuracy, precision, and recall. This algorithm can play a pivotal role in medical imaging for early and accurate brain tumor detection, offering clinicians more reliable tools for diagnosis. Additionally, the model’s success in glioma detection has the potential to be a breakthrough in clinical practice for more precise brain tumor identification.
[25]	Exploring AI’s role in public health research and scientific authorship	This study explores the potential of GPT-3, an advanced AI model, to support public health research by generating relevant text blocks and summarizing scientific articles. The research specifically tests GPT-3’s capacity to provide plausible answers on public health issues. Despite some fabricated content, it demonstrates the potential for AI to assist researchers in compiling and analyzing large amounts of data. It also discusses the implications of using AI as a scientific contributor in research publications while adhering to the principles of scientific integrity.	This study highlights the potential of AI to assist in public health research by summarizing the literature and generating scientific content. However, it also raises critical questions about the credibility of AI-generated information and its appropriate role in authorship. This research emphasizes the importance of establishing guidelines for AI’s involvement in scientific research, ensuring that its contributions are valid and adhere to ethical standards.
[26]	AI-powered COVID-19 CT image diagnosis and lesion segmentation	This study presents a deep learning-based method for accurately segmenting lesions from CT images of COVID-19 patients. The proposed solution, DLShelper, corrects rough initial labels of lesions by analyzing pixel distribution in infected and healthy areas. By improving segmentation accuracy, the system aids in diagnosing COVID-19 and grading the severity of lung lesions. The approach is tested on a public dataset of CT images and has been shown to significantly improve segmentation and diagnosis accuracy, benefiting medical staff with better diagnostic support.	The DLShelper tool offers a promising AI-driven solution for diagnosing COVID-19 through enhanced lesion segmentation and severity grading. It assists clinicians by providing more accurate and detailed diagnostic information, improving decision-making during the diagnosis process. The tool’s effectiveness in handling CT images can be adapted for other diagnostic applications, paving the way for more advanced, AI-powered diagnostic systems in healthcare.

**Table 2:** Summary of the reviews published in this Topic.

Study/Type	Focus	Brief Summary	Contribution
[27]/R	Artificial intelligence in cytopathology	This umbrella review delves into the integration of artificial intelligence (AI) into the field of cytopathology, highlighting its potential to improve diagnostic accuracy and operational efficiency. This study examines 21 review articles to identify key trends, opportunities, challenges, and best practices for AI adoption in cytopathology. It emphasizes the importance of validation processes, integration with existing clinical workflows, and addressing ethical issues such as algorithmic bias and data privacy.	This review contributes by offering an extensive analysis of the current landscape of AI in cytopathology, revealing how AI can automate diagnostic processes, reduce errors, and improve patient outcomes. It highlights critical challenges, such as high implementation costs, the need for empirical data on diagnostic accuracy, and the integration of AI with existing clinical systems. This review also provides a roadmap for future AI development in cytopathology, informed by advancements in related fields like histopathology and radiology.
[28]/SR	Generative Adversarial Networks (GANs) in head and neck surgery	The review explores the application of Generative Adversarial Networks (GANs) in the field of head and neck surgery. GANs are a class of AI algorithms capable of generating new data from existing data. This review examines how GANs are being used in diagnostic imaging and treatment planning in craniofacial surgery, particularly for conditions such as craniosynostosis, chronic sinusitis, and various bone defects. The review assesses 9 studies out of 700, summarizing 8 key applications of GANs in the head and neck region.	This study highlights the emerging role of GANs in improving diagnostic accuracy and treatment outcomes in head and neck surgery. It identifies several practical applications of GANs, including the enhancement of diagnostic imaging, surgical planning, and post-operative prediction. The review emphasizes GANs as a transformative tool in personalized healthcare, providing a more precise and data-driven approach to treating head and neck pathologies, including oncological cases.
[29]/R	Mobile and domiciliary radiology with AI integration	This review investigates the growing integration of mobile radiology and AI technologies in healthcare, particularly focusing on how these innovations have transformed domiciliary (home-based) radiology services. This study assesses 21 recent publications to analyze the effectiveness of mobile X-ray equipment, AI-enhanced diagnostic tools, and telemedicine applications, with a focus on the post-COVID-19 era. Notably, it explores how mobile mammography units have become essential for breast cancer screening in underserved populations.	The contribution of this review is in examining how AI, mobile radiology, and telemedicine technologies can significantly improve access to diagnostic services in rural and underserved areas. It also discusses the potential of AI in enhancing pediatric radiology and the broader implications for healthcare equity. The review offers insights into the future of domiciliary radiology, suggesting the importance of multi-domain technology assessment and regulatory frameworks for the responsible integration of these technologies.
[30]/SR	AI chatbots in women’s health	This systematic review and meta-analysis explores the use of AI-powered chatbots in improving health outcomes for women, specifically focusing on mental health (e.g., anxiety, depression), health behaviors (e.g., cancer self-care), and preconception care. By analyzing 10 studies published between 2019 and 2023, this review assesses the effectiveness of chatbot interventions in various health domains, including reproductive health, eating disorders, and relationship management.	This study underscores the positive impact AI chatbots can have on women’s health by providing accessible, digital therapeutic interventions. This review provides evidence that chatbots can improve mental health outcomes and health behaviors among women, offering a cost-effective and scalable solution for addressing issues such as anxiety, depression, and self-care practices. It emphasizes that chatbots could become a critical tool in healthcare interventions, especially for women in remote or underserved areas.
[31]/R	AI and language models in cardiology	This review examines the integration of generative AI, such as ChatGPT-4, and other language models in cardiology. It focuses on how AI can assist in diagnosing heart disease, recommending treatment plans, and improving physician efficiency through administrative task automation. It discusses the challenges associated with AI, such as outdated information, high costs, and the potential loss of human empathy in patient care.	This study explores the role of AI in revolutionizing cardiology by improving diagnostic accuracy, treatment planning, and operational efficiency. It highlights both the benefits of AI tools—such as enhancing physician decision-making and reducing administrative burdens—and the challenges, such as maintaining up-to-date knowledge and ensuring equitable access to these technologies. This review calls for comprehensive training for healthcare professionals and careful integration of AI into medical practices to ensure patient-centered care.
[32]/R	AI in teledermatology	This review discusses the role of AI in advancing teledermatology, particularly in diagnosing skin conditions remotely through telemedicine platforms. It evaluates the integration of AI in eHealth and mHealth for self-care, quality control, and patient management in dermatology. This study also addresses the challenges of app validation, standardization, cybersecurity, and the need for legal and regulatory frameworks.	This study highlights the potential of AI to improve the quality of care in dermatology by facilitating remote diagnosis and patient monitoring. It identifies key benefits such as increased accessibility, reduced healthcare costs, and improved efficiency in diagnosis. However, this review also emphasizes the need for enhanced app design, better cybersecurity measures, and standardized protocols for AI applications in teledermatology. It suggests that regulatory frameworks and position statements from scientific societies are critical to ensuring responsible implementation.
[33]/R	Machine learning in domestic violence detection	This review explores the use of machine learning (ML) algorithms in detecting and predicting domestic violence (DV) through the analysis of digital data, including social media, surveys, and health records. The study reviews 22 articles and identifies key applications of both supervised and unsupervised ML methods, focusing on their ability to classify, predict, and explore patterns associated with DV.	The contribution of this review lies in demonstrating the potential of machine learning techniques to identify early signs of domestic violence by analyzing digital text data. It provides insights into the algorithms used in this field, such as random forests and support vector machines, and discusses the challenges, including data quality, algorithmic bias, and the need for extensive data preparation. The review encourages further development of ML models specifically designed for DV detection, which could significantly enhance early intervention efforts.

**Table 3:** Summary of other studies published in this Topic.

Study/Type	Focus	Brief Summary	Contribution
[34]/TN	Machine learning for effective dose calculation	This study explores the use of machine learning (ML) algorithms to predict organ doses and effective dose conversion coefficients (DCCs) for various anthropomorphic phantoms. Traditionally, Monte Carlo methods are used for these calculations, which are time-consuming and require expertise. By compiling a comprehensive dataset from Monte Carlo studies, the authors train ML models (XGB, GB, and Extra Trees regressor) to predict the effective dose. This study evaluates the ML models’ performance using error metrics and compares them with ICRP values.	This study highlights the potential of ML techniques to predict effective doses efficiently, offering a solution to the traditional Monte Carlo method’s limitations. The ML models demonstrated good accuracy at mid-range neutron energies, providing a faster alternative for dose prediction, especially in scenarios requiring quick results. Additionally, this study suggests improvements in data representation and the inclusion of larger datasets to enhance model accuracy, thereby contributing to personalized dosimetry in radiation protection.
[35]/P	Neural network models for causal variable analysis in medical and climatic studies	This paper presents a neural network-based method to analyze non-linear systems, focusing on small datasets. The authors propose a strategy to identify the roles (linear, non-linear, or threshold) of causal variables in predicting the behavior of a target variable. They apply this method to climate studies and medical research, particularly cancer studies. The approach allows for predictions about future scenarios based on causal variables.	This paper introduces a complementary approach to deep learning by focusing on understanding causal relationships within datasets. The methodology has applications in medical research, particularly in cancer studies, by helping identify important causal variables and predicting future trends. The approach’s utility in small datasets makes it valuable for medical studies with limited data, and its potential to influence research on cancer treatment is a key contribution.
